# Exploring the influence of age, gender, stigma, and years living with HIV on mental health outcomes

**DOI:** 10.1111/hiv.70098

**Published:** 2025-08-19

**Authors:** Guilherme Welter Wendt, Lara Wiehe Chaves, Angelo Brandelli Costa

**Affiliations:** ^1^ Postgraduate Program in Applied Health Sciences Western Paraná State University Francisco Beltrão Brazil; ^2^ Graduate Program in Medicine and Health Sciences Pontifical Catholic University of Rio Grande do Sul Porto Alegre Brazil; ^3^ Department of Psychology, Graduate Program in Psychology and John Cabot University Pontifical Catholic University of Rio Grande do Sul Porto Alegre Brazil

**Keywords:** gender, HIV, mental health, risk factors, stigma

## Abstract

**Background:**

People living with HIV/AIDS face a myriad of discrimination and social stigma experiences. As a result of progress observed throughout the HIV epidemic, an ageing population of people living with HIV/AIDS exists, potentially facing greater mental health challenges from combined chronic conditions and stigma. Hence, this research aims to determine the additional value of age, years living with HIV, and gender, in conjunction with overall and internalized stigma in predicting clinically significant symptoms of depression and anxiety.

**Methods:**

The sample consists of 1666 people living with HIV PLHA, aged between 18 and 76 years who participated in a community‐based study across Brazil. Participants provided responses on HIV‐related stigma, Internalized AIDS‐Related Stigma, and to the Patient Health Questionnaire, which demonstrated excellent psychometric proprieties.

**Results:**

Gender and stigma increased the likelihood of significant symptoms of anxiety, accounting for the influence of age and years of living with HIV. Odds were higher among those who reported transgender identity (OR^a^ = 2.05; 95% CI: 1.13, 3.70). Also, women reported significantly higher chances for anxiety (OR^a^ = 1.36; 95% CI: 1.05, 1.76). Both HIV‐related (OR^a^ = 1.05; 95% CI: 1.01, 1.08) and internalized stigma (OR^a^ = 1.30; 95% CI: 1.21, 1.40) were associated with anxiety. General and internalized stigma were the unique predictors for depression, with adjusted OR ranging from 1.07 (95% CI: 1.03, 1.10) to 1.41 (95% CI: 1.31, 1.53), respectively.

**Conclusions:**

Stigma constitutes a significant obstacle for initiatives aimed at HIV prevention and therapeutic programmes, and the main findings of this study revealed that factors associated with clinically significant symptoms of depression and anxiety were predominantly allied with psychosocial stressors and gender identity indicators. Limitations, implications for practice and policy are addressed.

## INTRODUCTION

Research on the relationship between stigma and mental health among people living with HIV/AIDS has primarily focused on marginalized and vulnerable groups [1]. By definition, HIV‐related stigma is a social construct in which negative attitudes, feelings and beliefs towards HIV are expressed, encompassing various types, such as internalized, anticipated, perceived, enacted, externalized and structural stigma [2, 3]. HIV stigma causes significant harm due to gender roles, including women and transgender individuals through external and internalized prejudice [4, 5, 6], especially in settings where HIV is associated with promiscuity or moral failure [7]. Gay men have shown experiences of fear, guilt, isolation, loneliness and poor social support when dealing with HIV diagnosis, with links to psychological suffering such as depression, while heterosexual men may avoid care due to masculinity threats [8, 9].

An overemphasis on HIV‐related stigma within particular groups may distort the scientific comprehension of variables linked to HIV and its associated health consequences. To accurately capture the social determinants of health outcomes, evidence‐based interventions must take into consideration the broader context. Proponents of this perspective assert that health and behavioural tendencies are shaped by a multifaceted interplay of various factors, rather than a single, universal or predetermined cause [10, 11, 12]. For instance, research by Chambers et al. has underscored the necessity for research to guide policy and practice related to HIV in individuals of older age, considering the rising number of people in this age group living with the virus [11].

In addition to age and gender, other established factors like years living with HIV have demonstrated varying connections to mental health in people living with HIV/AIDS [9]. Newly diagnosed individuals face acute stigma linked to poorer viral suppression, while those with prolonged infection experience evolving challenges from ‘survivor's guilt' in long‐term survivors to assumptions about disease progression in advanced cases [13, 14]. Notably, interventions targeting these duration‐specific effects have demonstrated concurrent improvements in both mental health and immunological markers (CD4 count, viral load), revealing the bidirectional nature of psychosocial and biological factors [15]. These temporal patterns intersect with gender and age disparities that differentially impact mental health outcomes across the HIV care continuum. Consequently, the primary goal of this research can be most clearly summarized by initially examining the current state of knowledge in this area, specifically by reviewing data on depression and anxiety. Upon achieving the objective, a subsequent section details the psychological impacts experienced by people living with HIV/AIDS, suggesting research priorities and outlining their application in real‐world settings within these communities.

### Depression and anxiety symptoms in the context of HIV

Comorbid depression is a significant issue among individuals living with HIV/AIDS, with varying prevalence rates occurring across different vulnerable populations. Prevalence of depression varies significantly, from approximately 12.7% among women living with HIV to over 60% in particularly susceptible groups [16, 17], with adolescents and young adults showing particularly high vulnerability (29%–51% prevalence) [18, 19]. Older adults with HIV also face elevated risks, reporting depressive symptoms often linked to difficulties in cognitive functioning [20, 21]. Studies assessing depressive symptoms in people living with HIV/AIDS commonly use self‐reported measures. For instance, a systematic review of 60 studies found that the links with depressive symptoms are positive and consistent in both HIV‐related stigma and internalized stigma related to HIV [22].

The bidirectional relationship between depression and HIV outcomes is evident, with mental health symptoms reducing treatment adherence [23] while simultaneously, poor physical health exacerbates depressive symptoms. Although depression can impair long‐term care engagement by patients, it does not consistently correlate with hospitalization or mortality, suggesting its primary impact lies in treatment behaviours rather than disease progression [24]. Furthermore, studies highlight critical gaps in care, including the absence of approved mental health medications in some settings [16] and disparities between clinician and patient assessments [17].

Likewise, anxiety disorders are highly prevalent among people living with HIV/AIDS, with studies reporting median rates of 35%–45% across diverse populations, though specific subgroups exhibit even greater vulnerability [17, 24]. For instance, younger populations—particularly adolescents and young women—show higher rates than older adults, underscoring significant age‐related disparities [18, 25]. Evidence from screening measures for anxiety symptoms in people living with HIV/AIDS points to positive associations in terms of both general HIV‐related stigma as well as for internalized stigma [21, 26]. Importantly, the timing of assessment influences the intensity of symptoms: anxiety peaks shortly after diagnosis and remains elevated even during viral suppression, demonstrating its persistence throughout the care continuum [16, 20]. Some of the existing research indicates that HIV‐related stigma is more closely linked to depression than to anxiety symptoms, with both conditions often surpassing clinically significant benchmarks [17, 24]. Anxiety symptoms are primarily driven by health status worries, and clinical factors play a key role. Comorbidities, advanced HIV disease stages, and declining CD4 counts are all linked to higher anxiety [17, 24]. Younger people living with HIV/AIDS often experience exacerbated symptoms due to disclosure fears and HIV‐related neuropsychiatric effects, whereas older adults (≥70 years) face anxiety tied to ageism and declining health [18, 20].

Among people living with HIV/AIDS, subjective health perceptions are more strongly associated with anxiety symptoms than objective clinical measures, with those self‐rating their health as poor exhibiting significantly heightened anxiety [16]. Paradoxically, while anxious patients demonstrate better clinic attendance, they simultaneously exhibit higher rates of ART nonadherence, revealing complex behavioural dynamics in HIV management [18, 24]. Compounding this effect, structural barriers—including fragmented healthcare systems and inadequate clinician recognition of anxiety symptoms—systematically perpetuate mental health treatment gaps [17, 24].

### The role of age, gender, stigma and years of living with HIV on mental health outcomes

Evidence regarding mental health outcomes in people living with HIV has significantly contributed to developing targeted strategies to mitigate its adverse effects, including targeted campaigns and professional training initiatives. For example, recent studies examining different types of stigma in people living with HIV/AIDS have substantially increased the understanding of direct and indirect effects on depression [27, 28]. A systematic review of 83 studies linking stigma to depression revealed that a substantial proportion of research on this theme concerns HIV (*n* = 27), followed by investigations that explored the role of stigma in depression among sexual and gender minorities [22]. Internalizing stigma can cause psychological suffering to an extent of affecting one's motivation to follow treatment regimens [28]. Linked to adverse health outcomes are also broader forms of discrimination, encompassing experiences of racial and ethnic groups, along with general stigma associated with HIV, as well as specific characteristics of other key populations [29, 30, 31, 32].

Age may also have a significant influence on key indicators of psychological and physical health for people living with HIV/AIDS. There is evidence suggesting that younger patients struggle to adhere to strict treatment regimens while prolonged lives with HIV present unique challenges for both patients and healthcare staff, including ageism and sexual minority stress [33, 34]. Chambers et al. conducted a review of existing research to investigate the impact of aging on the health of individuals aging with HIV. The research comprised a total of 209 studies, including two systematic reviews, 174 quantitative studies, 28 qualitative studies and five mixed methods studies. The authors emphasized the need to consider not just the clinical characteristics of people living with HIV/AIDS, but also the diversity within the patient sample in their proposed guidelines [11].

This research aims to explore the additional value of age, years living with HIV, and gender, combined with general HIV‐related and internalized stigma, in predicting depression and anxiety symptoms. The study's significance can be viewed from a distinct perspective. Initially, the investigation will examine some of the most substantial risk factors connected to detrimental mental health results, specifically depression and anxiety in people living with HIV/AIDS. The research will likewise expand upon and supplement existing studies, examining whether the previously identified predictors of poorer mental health outcomes in people living with HIV/AIDS, as reported in prior research, remain statistically significant when controlling for the impact of stigmatizing experiences [20, 21].

## MATERIALS AND METHODS

### Study population and procedures

This community‐based study included the participation of 1,666 people living with HIV/AIDS, recruited from seven Brazilian capitals. The number of individuals followed a priori sample size calculation. Further details on demographics and clinical information on this population can be found in the Results section, as well as by consulting the Stigma Index 2.0 Executive Summary [35]. Under the coordination of researchers from the Pontifical Catholic University of Rio Grande do Sul, 30 people living with HIV/AIDS received training to conduct data collection. Complete details on data collection can be found elsewhere [35].

The research sample was selected using a non‐probabilistic snowball sampling method, starting with contacts from interviewers recruited in the seven major cities in Brazil. The group was encouraged to identify potential participants from their peer networks, as well as through support groups, testing sites, healthcare services, and organizations providing HIV/AIDS services. The selection of interviewees considered a diverse profile and the priorities of the Brazilian HIV/AIDS response, including representation of Black individuals, young people, elderly individuals and key populations. The proportion of responses was as follows: 26.41% from São Paulo, 14.71% from Rio de Janeiro, 14.35% from Porto Alegre, 14.23% from Recife, 14.11% from Salvador, 10.92% from Manaus and 5.28% from Brasília.

### Variables and measurement tools

The outcomes of this study include scores of anxiety and depression as measured by the Patient Health Questionnaires (PHQ‐4) [36]. The PHQ‐4 is a brief screening tool composed of two validated subscales: the PHQ‐2 for depression screening and the Generalized Anxiety Disorder screener (GAD‐2) for anxiety assessment [36]. The PHQ‐2 collects self‐reports of two core symptoms of depression (anhedonia and depressed mood), while the GAD‐2 assesses two cardinal symptoms of anxiety (nervousness and uncontrollable worry), with items derived from Diagnostic and Statistical Manual of Mental Disorders, Fourth Edition (DSM‐IV) criteria [36, 37]. Responses range from 0 ‘Not at all’ to 3 ‘Nearly every day’. For each subscale, values equal or above 3 suggests clinically significant symptoms (*α* = 0.82). Confirmatory Factor Analyses (CFA) analyses were conducted with Diagonally Weighted Least Squares estimator and by calculating Bentler‐Bonett normed fit index (NFI), Tucker‐Lewis index (TLI), comparative fit index (CFI), root mean square error of approximation (RMSEA). Goodness of fit (*χ* ^2^(1) = 1.64, *p* = 0.20) and fit indices for the PHQ‐4 were within the optimal range (CFI = 0.99; NFI = 0.99; RMSEA = 0.01; SRMR = 0.01; TLI = 0.99).

The independent variables were extracted from the Stigma Index 2.0 and that demonstrated theoretical and ecological relevance to the study (namely key demographic and HIV‐related information, as well as a measure of general stigma related to HIV [*α* = 0.79]). These all are part of a comprehensive list of questions that have been fully described previously [1]. Another measure of stigma—the Internalized Stigma Scale (IA‐RSS)—was used to measure one's unique perspective regarding experiences of HIV stigma [38]. This measure consists of 6 items that tap into the internalization of stigmas, including feelings of guilt and shame (*α* = 0.73). Responses are given in a dichotomous manner. Higher scores indicate higher internalized stigma [38]. The measure's construct validity is supported by its significant correlations with key psychosocial variables, including increased depression symptoms, diminished self‐esteem, and poorer treatment adherence behaviours among HIV‐positive individuals. Representative items from the IA‐RSS include statements assessing shame (e.g., ‘I feel ashamed of having HIV’), self‐worth (e.g., ‘Having HIV makes me feel like a bad person’), and social avoidance (e.g., ‘I avoid social situations because of my HIV status’). The measure has demonstrated utility across diverse demographic groups, including different gender and ethnic populations living with HIV [39]. For the IA‐RSS, indices from CFA were also calculated, with an overall model fit (*χ* ^2^(1) = 16.53, *p* = 0.02) and fit measures (CFI = 0.99; NFI = 0.99; RMSEA = 0.02; SRMR = 0.01; TLI = 0.99) suggesting adequate results.

### Data analyses and ethical aspects

Data were modelled in the *Statistical Package for the Social Sciences* (v. 25; Arlington, NY) and analyzed in the Jasp Program (v. 0.19; Amsterdam, NL). Analyses included descriptive statistical procedures, such as means (*μ*), standard deviations (SD), frequencies and percentages. Inferential techniques were performed according to the study's goal and included Mann–Whitney, chi‐square (*X* ^2^) and regression procedures (binary logistic regression, stepwise method), according to each type of analysis and assumption [40]. Following past research, multivariate analyses controlled for the effects of both independent variables and covariates of interest [41]. Moreover, the research followed all national and international protocols regarding ethics in research involving human beings. Participants signed Consent Forms and were reassured about the ethical aspects related to the research. Also, data collection took place only after ethical clearance (n. 99716918.5.0000.5336).

## RESULTS

In Table 1, descriptive statistics are presented with comparisons in terms of the presence of clinically significant symptoms of depression and anxiety according to this study's variables of interest. Most of the sample reported not being employed at the time of data collection (*n* = 862; 51.90%) or studying (*n* = 1236; 74.23%). 71.20% (*n* = 1184) did not have a higher education degree.

**TABLE 1:** Descriptive statistics, including frequencies and valid percentages for each of this study's main variables according to the presence of clinically significant symptoms of depression and anxiety.

	Total sample	Clinically significant symptoms of depression		Clinically significant symptoms of anxiety	
		Yes	No	Yes	No
Age (M ± SD)^a^	40.12 (13.01)	39.72 (12.76)	40.33 (13.16)	40.10 (12.95)	40.13 (13.09)
General HIV‐related stigma (M ± SD)^a^	23.02 (3.69)	**23.83 (3.86)**	**22.65 (3.55)**	**23.50 (3.73)**	**22.63 (3.62)**
Internalized HIV stigma (M ± SD)^a^	8.72 (1.71)	**9.44 (1.75)**	**8.34 (1.56)**	**9.17 (1.72)**	**8.30 (1.58)**
Years living with HIV (*n*; %)^b^					
Up to 1 year (acute infection)	203 (12.35)	74 (13.23)	129 (11.99)	110 (13.80)	93 (11.09)
>1 and less than 6 years (intermediate infection)	404 (24.55)	120 (21.47)	280 (26.02)	**168 (21.10)**	**232 (27.65)**
6 years or more (long‐term infection)	1038 (63.10)	365 (65.30)	667 (61.99)	519 (65.11)	514 (61.26)
Gender (*n*; %)^b^					
Trans/non‐binary	70 (4.26)	26 (4.63)	41 (3.82)	38 (4.75)	29 (3.47)
Male	1036 (63.02)	315 (56.15)	717 (66.82)	455 (56.94)	577 (69.10)
Female	538 (32.73)	**220 (39.22)**	**315 (29.35)**	**306 (38.30)**	**230 (27.52)**

*Note*: In bold, significant differences between groups.

Abbreviations: M, mean; SD, standard deviation.

^a^ Mann–Whitney tests.

^b^ *X* ^2^ tests.

Participants with clinically significant symptoms of depression exhibited higher general HIV‐related and internalized HIV stigma compared with those without clinically significant symptoms (effect sizes: 0.20 and 0.35). Also, higher HIV‐related and internalized stigma scores were found in those with clinically significant symptoms of anxiety (effect sizes: 0.16 and 0.29). Females showed a higher expected frequency for clinically significant symptoms of depression. For clinically significant symptoms of anxiety, individuals living with HIV for 1–6 years reported lower expected values, and females showed higher proportions (Table 1).

Regression analyses models took age and time living with HIV as covariates. As per Table 2, gender and stigma increased the likelihood of clinically significant symptoms of anxiety, accounting for the influence of age and years of living with HIV. The estimates were highest among transgenders, and women reported slightly lower estimates; general and internalized stigma were associated with clinically significant symptoms of anxiety. General and internalized stigma were the single predictors of clinically significant symptoms of depression.

**TABLE 2:** Final models showing the factors independently associated with clinically significant symptoms of depression and anxiety.

Outcome	OR^c^	95% CI		OR^a^	95% CI	
		Lower	Upper		Lower	Upper
Clinically significant symptoms of depression						
Age	1.001	0.988	1.013	‐	‐	‐
Time living with HIV	1.034	0.931	1.150	‐	‐	‐
Gender: Male (Ref)	1	‐	‐	‐	‐	‐
Gender: Female	1.245	0.938	1.165	‐	‐	‐
Gender: Trans and non‐binary	1.444	0.778	2.680	‐	‐	‐
General HIV‐related stigma	0.017	1.036	1.107	**1.071**	**1.036**	**1.107**
Internalized stigma	0.038	1.316	1.530	**1.419**	**1.316**	**1.530**
Clinically significant symptoms of anxiety						
Age	0.999	0.985	1.007	‐	‐	‐
Time living with HIV	1.072	0.972	1.183	‐	‐	‐
Gender: Male (Ref)	1	‐	‐	‐	‐	‐
Gender: Female	0.131	1.053	1.763	**1.363**	**1.053**	**1.763**
Gender: Trans and non‐binary	0.302	1.131	3.700	**2.045**	**1.131**	**3.700**
General HIV‐related stigma	0.016	1.018	1.086	**1.052**	**1.018**	**1.086**
Internalized stigma	0.036	1.217	1.401	**1.306**	**1.217**	**1.401**

*Note*: OR^a^ = adjusted OR for all the other independent variables in the model; OR^c^ = crude OR. In bold, significant findings.

Abbreviations: CI, confidence interval; OR, odds ratio.

## DISCUSSION

Psychological research shows that the internalization of social norms and the human tendency to conform demonstrate stigma's significant influence on personal beliefs. This understanding is relevant in health settings like the HIV outbreak, where prejudice and exclusion have negatively impacted mental health to varying degrees across different groups [30]. In this respect, the goal of this study was to examine the additional value of age, years of living with HIV, and gender, in conjunction with overall and internalized stigma in predicting clinically significant symptoms of depression and anxiety in a representative sample of people living with HIV/AIDS in Brazil.

Research into the effects of age on a perso's overall well‐being is becoming more significant in patient care. This is due to the fact that HIV infection is now a long‐term condition. While some research has been conducted, only a few studies have controlled their analyses by participant's age and duration since diagnosis when exploring mental health outcomes [42]. This research built upon previous findings, considering the impact of age and the experiences of stigmatization as reported by the individuals involved.

Despite the significant gender differences found in bivariate analyses, where women had higher expected frequencies for clinically significant symptoms of depression, our research highlights the importance of general and internalized stigma in the context of depression. As shown in Table 2, these variables comprised the unique predictors of clinically significant depressive symptoms. In other words, a one‐unit increase in the individual's responses to general HIV‐related stigma resulted in a 7% increase (95% CI: 1.03, 1.10) for clinically significant symptoms of depression. With even higher intensity, internalized stigma resulted in a probability 41% higher to present with clinically significant symptoms of depression (95% CI: 1.31, 1.53).

Results on depression remained statistically significant even after accounting for the effects of age, gender and years of living with HIV. According to McGowan et al., depression is prevalent among individuals with HIV/AIDS, and they also noted that despite the notion that older people with HIV are more susceptible to depression due to stigma and limited social support, the relationship between age and psychological symptoms among people with HIV remains unclear [42]. The interconnection between age, mental distress and people living with HIV/AIDS may be closely linked to other relevant considerations, including quality of life (QoL). This approach might operate as a proxy for a multifaceted construct, thus encompassing physical, emotional, mental, social and behavioural aspects.

Comparing results directly with other studies requires caution due to the use of different measurement methods and varying predictors and covariates in the models. The current Stigma Index 2.0 is a relatively recent development, with limited research available that examines the same collection of variables being studied in this investigation. Nonetheless, the adjusted estimates from regression analyses resemble findings from other studies with diverse backgrounds. For example, Mwangala and collaborators reported an OR of 1.68 (95% CI: 0.84, 3.38) for the female category when predicting depressive symptoms [21]. These results indicated that being a woman did not increase the risks for clinically significant symptoms of depression, just like this study has found (Table 2). In terms of anxiety symptoms, a summary of the main findings shows that gender and stigma increased the likelihood of clinically significant symptoms—even after accounting for the effect of age and years living with HIV. The estimates were higher among transgenders, followed by women, who reported slightly lower estimates. For comparison, our findings provide support for Reisner et al.'s (2023) study, which documented statistically significant differences in clinically significant symptoms of anxiety, as measured by the PHQ, among transgender women compared with cisgender men and women. For symptoms of depression, scores did not differ between groups [43]. Evidence from a meta‐analytical study including 7,602 adult women with HIV/AIDS partially corroborated the findings regarding depression and anxiety symptoms. While authors argued that symptoms of depression and anxiety in women appear equivalent in terms of effect size estimates, our investigation only found anxiety symptoms to be associated with female gender [44].

In line with previous research, internalized stigma increased the risks for negative mental health outcomes [45, 46, 47]. For the two outcomes, both general and internalized HIV stigma were associated with clinically significant symptoms of anxiety in the final, adjusted predictive model depicted in Table 2. The estimates for the depression were slightly higher, in which general and internalized stigma were the unique predictors. Strikingly, internalized stigma was found to be a substantial predictor in both models. These results add to a quite abundant literature linking HIV to depression and anxiety [48, 49] while also claiming attention to psychosocial stressors that negatively impact those living with HIV in Brazil. Indeed, stigma appears to be crucial in determining the quality of one's mental health. For PLHA, with HIV‐related stigma consistently emerging as a key predictor across studies, significantly increasing odds of depressive and anxiety symptoms [19, 21, 25]. Rueda et al. (2016), in a series of meta‐analyses, reported consistent associations between HIV‐related stigma and higher anxiety and depression symptoms across various settings and populations [50].

People living with HIV/AIDS experience numerous forms of stigma and prejudice throughout their lives. These encompass difficulties experienced with families, social circles, close relationships and medical caregivers [51]. Furthermore, people living with HIV/AIDS often encounter disrespectful treatment, accompanied by infringements on their rights within establishments, organizations and other environments. Due to these overlapping challenges, people living with HIV/AIDS may resort to isolation as a way to shield themselves from traumatic and hurtful circumstances [4].

The results reported here may be explained by this. Individuals living with HIV/AIDS who face stigma and additional stressors related to their gender identity may exhibit social and emotional withdrawal as a potential coping mechanism; although this behaviour is unfortunately associated with lower adherence to treatment [9, 52], either in the forms of missing medications due to fear and preoccupations of having the diagnosis revealed without consent, delays in starting HIV treatment since a diagnosis is confirmed, along with disengagement with health professionals [4, 5, 6].

The hypothesis that age could be associated with clinically significant symptoms of depression in the sample was not supported by the data. This contradicts some of the existing literature indicating that age can have a significant impact on key indicators of both psychological and physical health in people living with HIV/AIDS. Research suggests that younger individuals with HIV may struggle to adhere to strict treatment protocols, and extended periods of living with the virus can present unique challenges for both patients and healthcare professionals [33, 34]. The data provided does not substantiate this claim. Potential explanations encompass the assessment tool used (i.e., PHQ‐4) and societal and cultural influences. Studies labelled as ‘contractive’ highlight the complexity of mental health issues affecting individuals with HIV/AIDS. Moreover, literature pointing to the potential role of years living with HIV and higher severity of depression and anxiety symptoms was only supported in bivariate analyses. When all the independent variables were taken into consideration, the length of HIV infection lost its statistical significance [18, 20].

In summary, significant harm is caused by HIV stigma resulting from societal gender roles, which disproportionately affect women through both external and internalized prejudice [4, 5, 6]. Excessive emphasis on stigma within specific groups can compromise the scientific comprehension of factors associated with HIV and its accompanying health outcomes. Evidence‐based interventions must take the broader context into account in order to accurately capture the social factors influencing health outcomes. Proponents of this perspective argue that health and behavioural patterns are shaped by a multifaceted dynamic, rather than a single, overarching, or predetermined cause [11, 12]. As a result, this research yields both direct and indirect implications for healthcare providers and policymakers, underscoring the significance of providing immediate psychological and social support to prevent suffering from stigmatizing experiences from escalating to critical levels [34, 45, 46, 49]. Such attitudes could assist in achieving the proposed goals for HIV elimination [53]. Addressing the various forms of stigma experienced by individuals living with HIV requires a comprehensive approach that encompasses psychological, social, behavioural, and public health factors, aligning with current competencies that are effective in HIV practice [45]. Moreover, it seems vital to deploy strategies that can successfully enhance social support and psychological well‐being. However, the unfortunate truth is that in most instances, significant strides in this area are rarely achieved. Social security systems, such as those that provide livelihood interventions to promote economic empowerment, can also help alleviate both the independent and dependent factors examined in this context [46].

### Limitations and further directions

In light of the inherent difficulty of fully grasping another person's experience, this study delved deeper into the domain of social determinants of mental health in individuals with people living with HIV/AIDS, with the aim of bridging the knowledge gaps in existing research and countering the detrimental impacts of stigma [42, 54]. Biomedical breakthroughs have significantly improved the prognosis of HIV, transforming it from a typically fatal condition to a manageable chronic illness; yet societal obstacles and stigma still impact people living with HIV/AIDS [1, 55].

More broadly, interventions that aim to eliminate stigma at the individual, community and structural levels are crucial. Findings such as those reported here are pivotal to the development of effective programs and policies for people living with HIV/AIDS. Readers should note the limitations associated with observational designs and the reliance on potential bias that might have occurred during data collection (i.e., desirability bias). In addition, it appears essential to stress that the sample, as representative and statistically powerful as it might be, could not provide a comprehensive and exhaustive picture of the Brazilian population living with HIV/AIDS.

Additionally, the PHQ‐4, which is used for screening purposes, may not fully capture all the symptoms of anxiety and depression. The PHQ‐4 may be beneficial in identifying those most vulnerable to psychological distress. Nonetheless, one of the strengths of this study resides in its description of excellent psychometric indices for the PHQ‐4, based on a large and nationally representative sample. Once again, implications for policy and practice emerge, which could be seen in the routine use of ultra‐short instruments like the PHQ‐4 in follow‐up consultations. Taking prevention and interventive actions at early stages could potentially lower the costs associated with referring patients from service to service. The study's findings also indicate further steps, such as research into other types of stigmas, including anticipated stigma. Longitudinal studies are also recommended to investigate the causal relationship between the variables that were linked to psychological distress in this sample.

## CONCLUSION

The singularity associated with how people living with HIV/AIDS internalize societal stigma related to HIV is profoundly complex; however, the implications of harbouring negative perceptions towards these individuals have substantially influenced their lives in and outside healthcare centres. This may potentially result in a recurring pattern of detrimental behaviours characterized by diminished self‐worth, feelings of guilt, shame and social withdrawal. The internalization of stigma by people living with HIV/AIDS constitutes a significant obstacle for both initiatives aimed at HIV prevention and therapeutic programmes, and the main findings of this study revealed that factors associated with depression and anxiety were predominantly allied with psychosocial stressors and gender identity indicators.

## AUTHOR CONTRIBUTIONS

Guilherme Welter Wendt, Lara Wiehe Chaves and Angelo Brandelli Costa were involved in the conceptualization and planning of the manuscript. Lara Wiehe Chaves and Guilherme Welter Wendt were responsible for the literature review, preliminary draft and revision of the manuscript. Angelo Brandelli Costa was involved in reviewing the manuscript, contributing legal and ethical considerations. All authors were involved in subsequent revisions of the manuscript.

## CONFLICT OF INTEREST STATEMENT

The authors declare no conflicts of interest.

## Data Availability

The data that support the findings of this study are available on request from the corresponding author. The data are not publicly available due to privacy or ethical restrictions.
